# Prevalence of *Plasmodium falciparum* infection among pregnant women at first antenatal visit in post-Ebola Monrovia, Liberia

**DOI:** 10.1186/s12936-018-2506-z

**Published:** 2018-10-11

**Authors:** Guillermo Martínez-Pérez, Dawoh Peter Lansana, Senga Omeonga, Himanshu Gupta, Bondey Breeze-Barry, Raquel González, Azucena Bardají, Adelaida Sarukhan, James D. K. Goteh, Edith Tody, Pau Cisteró, Benard Benda, Juwe D. Kercula, Fanta D. Kibungu, Ana Meyer García-Sípido, Quique Bassat, Christine K. Tarr-Attia, Alfredo Mayor

**Affiliations:** 1 ISGlobal, Hospital Clínic-Universitat de Barcelona, Barcelona, Spain; 2 Saint Joseph’s Catholic Hospital, Oldest Congo Town, PO Box 10-512, 1100 Monrovia, Liberia; 3 Liberia Medicines and Health Products Regulatory Authority, Monrovia, Liberia; 4 NGO Juan Ciudad Foundation, Madrid, Spain; 5 Centro de Investigação em Saúde de Manhiça (CISM), Maputo, Mozambique; 6 ICREA, Pg. Lluís Companys 23, 08010 Barcelona, Spain; 7 Pediatrics Infectious Diseases Unit, Pediatrics Department, Hospital Sant Joan de Déu (University of Barcelona), Barcelona, Spain

**Keywords:** Malaria, Pregnancy, Prevalence, Antenatal, Liberia, Parasite resistance

## Abstract

**Background:**

Disruption of malaria control strategies during the West African 2014–2016 Ebola epidemic led to an increase in malaria-attributable mortality. However, recent data on malaria infection in vulnerable groups, such as pregnant women, are lacking in this post-Ebola scenario. This cross-sectional study aimed to assess the prevalence of *Plasmodium falciparum* infection and of molecular markers of drug resistance among pregnant women attending antenatal care in Monrovia, capital of Liberia.

**Methods:**

From October 2016 to June 2017, all pregnant women attending their first antenatal care visit at the Saint Joseph’s Catholic Hospital, Monrovia, were invited to participate in the study. In addition to their routine antenatal care tests, capillary blood spotted onto filter papers were collected from all consenting participants to determine presence of *P. falciparum* by real-time quantitative PCR. Molecular markers of anti-malarial drug resistance were assessed through Sanger sequencing and quantitative PCR in specimens positive for *P. falciparum* analysis.

**Results:**

Of the 195 women participants, 24 (12.3%) were *P. falciparum*-positive by qPCR. Infected women tended to be more commonly primigravidae and younger than uninfected ones. Parasite densities were higher in primigravidae. Fever was more frequently detected among the infected women. No statistically significant association between *P. falciparum* infection and haemoglobin levels or insecticide-treated net use was found. While high prevalence of genetic polymorphisms associated with chloroquine and amodiaquine resistance were detected, no molecular markers of artemisinin resistance were observed.

**Conclusion:**

*Plasmodium falciparum* infections are expected to occur in at least one in every eight women attending first ANC at private clinics in Monrovia and outside the peak of the rainy season. Young primigravidae are at increased risk of *P. falciparum* infection. Molecular analyses did not provide evidence of resistance to artemisinins among the *P. falciparum* isolates tested. Further epidemiological studies involving pregnant women are necessary to describe the risk of malaria in this highly susceptible group outside Monrovia, as well as to closely monitor the emergence of resistance to anti-malarials, as recommended by the Liberian National Malaria Control Programme.

## Background

Children and pregnant women are particularly vulnerable to *Plasmodium* spp. infection and disease, which translates into significant negative consequences for the health of mothers and infants [1–3]. In Liberia, West Africa, malaria transmission is perennial throughout the year, and infections are mostly caused by *Plasmodium falciparum* [4]. According to the 2011 and 2016 National Malaria Indicator Surveys (MIS), malaria prevalence remains high, peaking at 45.0% among children 6–59 months of age [5, 6].

The Liberian Ministry of Health and Social Welfare (LMoHSW) recommends the use of artemisinin-based combined therapy (ACT), intermittent preventive treatment during pregnancy (IPTp) with sulfadoxine–pyrimethamine (SP), and provision of insecticide-treated nets (ITN) as key strategies for control of malaria in pregnancy [7]. As in other sub-Saharan African countries, uptake of IPTp remains suboptimal and far from global coverage targets [8]. The 2013 national Demographic and Health Survey (DHS) reported that 48% of women who gave birth in the past 2 years had received two or more doses of IPTp-SP and that 37% of pregnant women slept under an ITN the preceding night [9].

Both the scale-up of ITN distribution and IPTp-SP, as strategies to protect pregnant women from malaria, were discontinued during the recent Ebola epidemic that resulted in over 10,000 deaths in Liberia, Sierra Leone and Guinea Conakry [10–12]. Mathematical modelling estimated that the interruption of malaria control could have caused a 62% increase in mortality by malaria [13]. Pregnant women were particularly affected since many stopped attending antenatal care (ANC) services and preferred to deliver outside the health facilities [14–16]. Fear of becoming infected with Ebola and the decimation of the healthcare workforce to this disease, in addition with the interruption of regular healthcare services added to the financial, geographical and social challenges that women were already facing to access healthcare in Liberia [11, 17].

Although progress has been made globally in the implementation of vector control tools and other preventive tools to decrease the incidence of malaria [18, 19], development of insecticide resistance from the vectors and of anti-malarial resistance from the parasite might hamper the success of available treatment and preventive tools [20, 21]. The real threat posed by the potential emergence and/or spread of malaria parasites resistant to artemisinin and its derivatives [22], and the need to guide the choice of anti-malarial drugs by National Malaria Control Programmes, all warrant enhanced molecular surveillance to map the prevalence of molecular markers of resistance, particularly in the absence of regional capacities to conduct in vivo clinical trials [23, 24]. Markers *k13* [25–27], *pfcrt* [28–33], *pfmdr1* [31, 34–37] and *pfdhps* [38] are of special interest to Liberia as—since the availability of substandard anti-malarials in the Liberian market is documented [39]—they could help monitor resistance to current LMoHSW-endorsed IPTp-SP and artesunate–amodiaquine-based first-line ACT [7].

To date, there is a dearth of studies assessing how interruption of malaria control and care services during the Ebola outbreak might have led to self-treatment with substandard anti-malarial drugs bought in local chemists and, as consequence, fuel the appearance of markers of parasite resistances. In addition, there are no available survey-based data on prevalence of *Plasmodium* spp. infection among pregnant women [5, 6] before and after the Ebola outbreak. To inform the design and roll out of enhanced malaria prevention and care targeting pregnant women in post-Ebola Liberia, baseline descriptive epidemiological data on prevalence of malaria infection and disease, as well as on the emergence of drug resistance are needed.

## Methods

### Aim and design

This was an observational cross-sectional study that aimed to assess the burden of malaria among pregnant women attending ANC in Monrovia. Specific objectives of the study included determining the prevalence of *P. falciparum* infection in this particular vulnerable group through molecular assays, and assessing molecular markers of drug resistance in *P. falciparum* isolates.

### Study site and population

The study was conducted at the Outpatients Department (OPD) of the not-for-profit Saint Joseph’s Catholic Hospital (SJCH) in Congo Town neighbourhood, Monrovia. The SJCH was founded in 1963 by the Hospitaller Order of the Brothers of St John of God. In 2014, the SJCH closed for 4 months after nine of its staff members died to Ebola. Since its reopening in 2015, the SJCH provides general services to the population of Monrovia. Although the SJCH applies a cost recovery system for the general public, the institution has a charity arm to subsidize healthcare-related costs for the most deprived ones. Its official fee for a first ANC is of 5.00 USD. However, as the SJCH is supported by the National HIV and Malaria Programmes, diagnostics and therapeutics for HIV/AIDS and malaria are provided free-of-charge.

Inclusion criteria set for sampling eligible participants at the OPD included: pregnant women of any gestational age attending ANC at the SJCH for the first time in their current pregnancy. Women unwilling to give consent to participate or who reported having received any IPTp dose during their current pregnancy were excluded. Calculations indicated that a sample of 198 women was needed to detect a prevalence of PCR-confirmed parasitaemia of 50% (conservative estimate) with a ± 5% precision and a confidence level of 95%.

### Recruitment and specimen collection

After an initial period of training to the SJCH staff, recruitment and specimen collection was done between October 2016 and June 2017. All women arriving at the OPD who met the inclusion criteria were invited to participate and informed of the study objectives and specimen collection procedures.

After providing written informed consent, they were queried regarding basic socio-demographic and malaria prevention-related data (i.e. use of ITN the preceding night, or last time their house was sprayed indoor-residual insecticides). Participants were also asked to volunteer their HIV and tuberculosis status, if known. An infrared thermometer was used to measure forehead temperature. Gestational age was assessed by date of last menstrual period and by measurement of fundal height. All data were manually captured by the recruiting midwife using individual standardized case report forms.

All participants were referred to the SJCH Laboratory for specimen collection. Results of routine tests done at the SJCH laboratory as part of standard ANC care for all pregnant women (i.e. HIV, haemoglobin and syphilis) were also captured in their case report forms. Haemoglobin level was obtained using point-of-care Hemocue Hb 301 (^®^Radiometer Group, Ängelholm, Sweden). HIV serostatus and syphilis screening were assessed using rapid tests SD Bioline HIV-1/2 3.0 (^®^Alere Inc., Waltham, MA) and SD Bioline HIV/Syphilis Duo (^®^Alere Inc., Waltham, MA), respectively.

### Laboratory methods

Capillary blood from the women’s fingers was collected on two Whatman 903 filter papers that were shipped to ISGlobal Laboratory (Barcelona, Spain) for molecular testing. The presence of *P. falciparum* was determined by real-time Quantitative PCR (qPCR). Briefly, DNA extracted by Chelex method from the filter paper was used for detection of 18S rRNA in duplicate [40, 41]. Parasite density was quantified by extrapolation of cycle thresholds (Ct) from a standard curve of *P. falciparum* ring-infected erythrocytes. A negative control with no template DNA was run in all reactions.

Samples that tested positive for *P. falciparum* were subjected for the analysis of molecular markers of anti-malarial drug resistance using previously described methods [42–44]. DNA from samples with less than 5 parasites/μL was subjected to pre-amplification using PicoPLEX WGA Kit (^®^Rubicon Genomics: Cat. No. —R30050) as per manufacturer’s instructions. DNA templates were then subjected to PCR amplification of molecular markers of *k13*, *pfcrt*, *pfmdr1* and *pfdhps* genes using a 2720 thermal cycler (^®^Applied Biosystems) followed by direct DNA Sanger sequencing bi-directionally [44]. A set of primer pair (Fw: caaattctatagtgtagttc; Rv: aattgtgtgatttgtccacaa) was used to sequence *pfdhps* 522–646 amino acid positions. For *pfpm2* and *pfmdr1* genes, copy number (CN) was also determined by SYBR-based qPCR (2× Power SYBR Green PCT Master Mix; ^®^Thermo Fisher Scientific, Washington, WA, UK) [44, 45]. All samples with estimated CN > 1.5 were repeated for confirmation. Water and human genomic DNA were used as negative controls. A total of six positive controls with known *k13* alleles; four parasite lines (3D7, 7G8, Dd2 and V1/S) with known *pfcrt* and *pfmdr1* alleles; four plasmids (DHPS-V1S, DHPS-PERU, DHPS-MALI and DHPS-DD2) with known *pfdhps* alleles; one positive control (C4) with known copies (3–4) of *pfpm2* gene; another positive control (Dd2) with known copies (3–4) of *pfmdr1* were also processed, amplified and sequenced. Isolates with mixed alleles were considered as mutated for the purposes of polymorphism frequency estimation.

### Data management

A unique identification number was used to link signed consent forms to case report forms. Case report forms did not include personal identifiers and were used to collect socio-demographic, malaria care-related data, and ANC routine laboratory tests data. Data from the case report forms were digitized by a SJCH trained clerk into an online case record form manager built in open source software OpenClinica (^®^OpenClinica LLL., Waltham, MA) and kept safely in a locked cabinet at the SJCH. ISGlobal Laboratory data were directly captured by the laboratory technician into the OpenClinica-generated dataset. Access to OpenClinica was password protected. OpenClinica server is kept at the Hospital Clínic (Barcelona, Spain) server.

### Definitions and statistical analysis

All blood specimens collected were included in the qPCR analysis. A positive *P. falciparum* infection in peripheral blood by qPCR, irrespective of parasite density, was the primary outcome in the analysis presented in this paper. Fever was defined as temperature ≥ 38 °C, and maternal anaemia as haemoglobin levels < 11 mg/dL (being mild if between 10 and 10.9 mg/dL, moderate if between 7 and 9.9 mg/dL, and severe if < 7 mg/dL). Women were classified as primigravidae (first pregnancy) or multigravidae (at least one previous pregnancy).

Cross tabulations and t-test, Pearson Chi squared test (χ^2^) or Fisher’s exact test were used, where appropriate, to explore whether socio-demographics and other health-related variables of interest were associated with *P. falciparum* infection. Data analysis was conducted using STATA v.12.0 (^®^StataCorp LLD., College Station, Texas, USA). Significance level was set at p < 0.05.

### Ethics

The study was conducted in accordance with the ICH E6 Good Clinical Practice [46], and under the provisions of the Declaration of Helsinki [47], and local rules and regulations. Written informed consent was sought for all women if 18 years of age or older. Parental consent in addition to minor assent was sought for all women aged younger than 18 years. Participants did not receive any retribution for their engagement as study subjects. Refusal to participate in this study did not affect service provision as per standard ANC practice. All women invited to enrol in this study, both consenting and non-consenting, received their first dosage of IPTp and treatment for anaemia, if necessary.

This research protocol was approved by the local University of Liberia-Pacific Institute Research and Evaluation Institutional Review Board (UL-PIRE, Monrovia, Liberia) and by the Hospital Clínic Health Research Ethics Committee (CEIC, Barcelona, Spain).

## Results

One hundred and ninety-eight women meeting inclusion criteria were invited to participate, and all of them consented. Of the 198 women enrolled, three decided to discontinue their participation before collection of blood specimens. The analysis presented is based on data from the 195 women with peripheral blood samples available and analysed for *P. falciparum* infection.

### Clinical and socio-demographics characteristics of study women

All participants were residents of Monrovia and peri-urban areas. Their mean age was 27.3 years (standard deviation [SD] 6.1; range 14–43) and 7 (3.6%) were younger than 18 years of age. More than half (57.9%) of the participants had concluded secondary studies and 15% had not completed any education (Table 1). One in four women (26.7%) were students with no source of income, whilst one third of the total (34.9%) were self-employed in the sales and services sector.

**Table 1:** Clinical and socio-demographic profile of study women

Variables	*n* (%)
Age (in years) range (*n *= 191)	
≤ 17	7 (3.7%)
18–25	65 (34.0%)
26–35	92 (48.2%)
≥ 36	27 (14.1%)
Mean (SD)	27.3 (6.10)
Anaemia (*n * = 193)	
Normal range (≥ 11 mg/dL)	113 (58.5%)
Anaemia (< 11 mg/dL)	80 (41.5%)
Mean haemoglobin levels (SD)	11.2 (1.42)
Gravidity (*n* = 194)	
Primigravidae	141 (27.3%)
Multigravidae	53 (72.7%)
Gestational age (*n *= 186)	
Mean (SD)	17.7 (7.14)
Fever (*n *= 188)	
No	182 (96.8%)
Yes, ≥ 38 °C at point of enrolment	6 (3.2%)
Anti-malarial use (*n * = 195)	
Not in the current pregnancy	140 (71.8%)
Yes in the current pregnancy	55 (28.2%)
Tuberculosis (*n * = 195)	
Not on TB treatment	193 (99.0%)
Self-reported on TB treatment	2 (1.0%)
HIV (*n *= 167)	
Confirmed negative test	162 (97.0%)
Confirmed positive test	5 (3.0%)
Syphilis (*n *= 160)	
Confirmed negative test	159 (99.4%)
Confirmed positive test	1 (0.6%)
ITN (*n *= 195)	
Did not use an ITN the preceding night	139 (71.3%)
Slept under an ITN the preceding night	56 (28.7%)
Education completed (*n * = 186)	
None	25 (14.6%)
Primary	24 (14.0%)
Secondary	97 (56.7%)
Tertiary/college	1 (0.6%)
University	17 (9.9%)
Current occupation (*n * = 194)	
Professional/technological	12 (7.0%)
Sales/services: self-employed	62 (36.3%)
Sales/services: salaried	12 (7.0%)
Skilled/unskilled manual	14 (8.2%)
Clerical	4 (2.3%)
Student (no other paid occupation)	43 (25.2%)
Unemployed	23 (13.5%)

*n* number of participants

Over a fourth (27.3%) of the participants were primigravidae (Table 1). Multigravidae women (*n* = 141) reported an average of 3.5 (range 2–10) pregnancies. Mean gestational age at first ANC visit was 17 weeks (SD 7.14).

Of the 193 women with haemoglobin test results, 29 (14.9%) and 51 (26.1%) had moderate and mild anaemia, respectively. No severe anaemia cases were detected. Six women (3.2%) had fever, and 55 (28.2%) self-reported having had taken anti-malarials in their current pregnancy.

Only two participants (1%) reported being on treatment for tuberculosis. No participant reported being HIV-infected. Of the 167 women who received an HIV rapid test, five (3.0%) had a confirmatory positive result. Among the 160 women who agreed to test for syphilis, only one (0.6%) was positive (Table 2).

**Table 2:** Health-related data and laboratory examinations

Variables	Uninfected (*n * = 171, 87.7%)^a^	*P. falciparum infection* (*n * = 24, 12.3%)^a^	p
	*n* (%)	*n* (%)	
Age (*n * = 191)			
Mean (SD)	27.6 (SD 5.88)	24.9 (7.19)	0.045^b^
Anaemia (*n * = 193)			
Mean haemoglobin levels (SD)	11.26 (1.45)	10.89 (1.13)	0.231^b^
Gravidity (*n * = 194)			
Multigravidae	128 (75.3%)	13 (54.2%)	
Primigravidae	42 (24.7%)	11 (45.8%)	0.030^c^
Gestational age (*n * = 195)			
1st trimester (1–14 weeks)	59 (34.5%)	10 (41.7%)	
2nd trimester (15–28 weeks)	100 (58.5%)	11 (45.8%)	
3rd trimester (≥ 29 weeks)	12 (7.0%)	3 (12.5%)	0.423^c^
Fever (*n * = 188)			
No	164 (98.2%)	18 (85.7%)	
Yes, ≥ 38 °C at point of enrolment	3 (1.8%)	3 (14.3%)	0.019^d^
Anti-malarial use (*n * = 195)			
Not in the current pregnancy	123 (71.9%)	17 (70.8%)	
Yes in the current pregnancy	48 (28.1%)	7 (29.2%)	0.911^c^
Tuberculosis (*n * = 195)			
Not on TB treatment	170 (99.4%)	23 (95.8%)	
Self-reported on TB treatment	1 (0.6%)	1 (4.2%)	0.232^d^
HIV rapid test (*n * = 167)			
Confirmed negative test	143 (97.9%)	19 (90.5%)	
Confirmed positive test	3 (2.0%)	2 (9.5%)	0.119^d^
Syphilis rapid test (*n *= 160)			
Confirmed negative test	140 (100.0%)	19 (95.0%)	
Confirmed positive test	0 (0.0%)	1 (5.0%)	0.125^d^
ITN (*n * = 195)			
Did not used an ITN the preceding night	123 (71.9%)	16 (66.7%)	
Slept under an ITN the preceding night	48 (28.1%)	8 (33.3%)	0.594^c^
Education completed (*n * = 186)			
None	25 (14.6%)	3 (12.5%)	
Primary	24 (14.0%)	2 (8.3%)	
Secondary	97 (56.7%)	16 (66.7%)	
Tertiary	1 (0.6%)	0 (0.0%)	
University	17 (9.9%)	1 (4.2%)	0.800^c^
Current occupation (*n * = 194)			
Professional/technological	12 (7.0%)	2 (8.3%)	
Sales/services: self-employed	62 (36.3%)	6 (25.0%)	
Sales/services: salaried	12 (7.0%)	2 (8.3%)	
Skilled/unskilled manual	14 (8.2%)	1 (4.2%)	
Clerical	4 (2.3%)	0 (0.0%)	
Student (no other paid occupation)	43 (25.2%)	9 (37.5%)	
Unemployed	23 (13.5%)	4 (16.7%)	0.420^c^

*n* number of participants

^a^Of the total (*n* = 195). Frequencies and rates in bivariate analyses below exclude missing values

^b^t-test

^c^Pearson χ^2^

^d^Fisher’s exact

All women but two (98.9%) reported that their home was never targeted in an indoor residual spraying program. Two-thirds of the total (71.3%) reported that they had not slept under an ITN the preceding night.

### Prevalence of *Plasmodium falciparum* infection and association with demographic and health-related variables

**Fig. 1:**
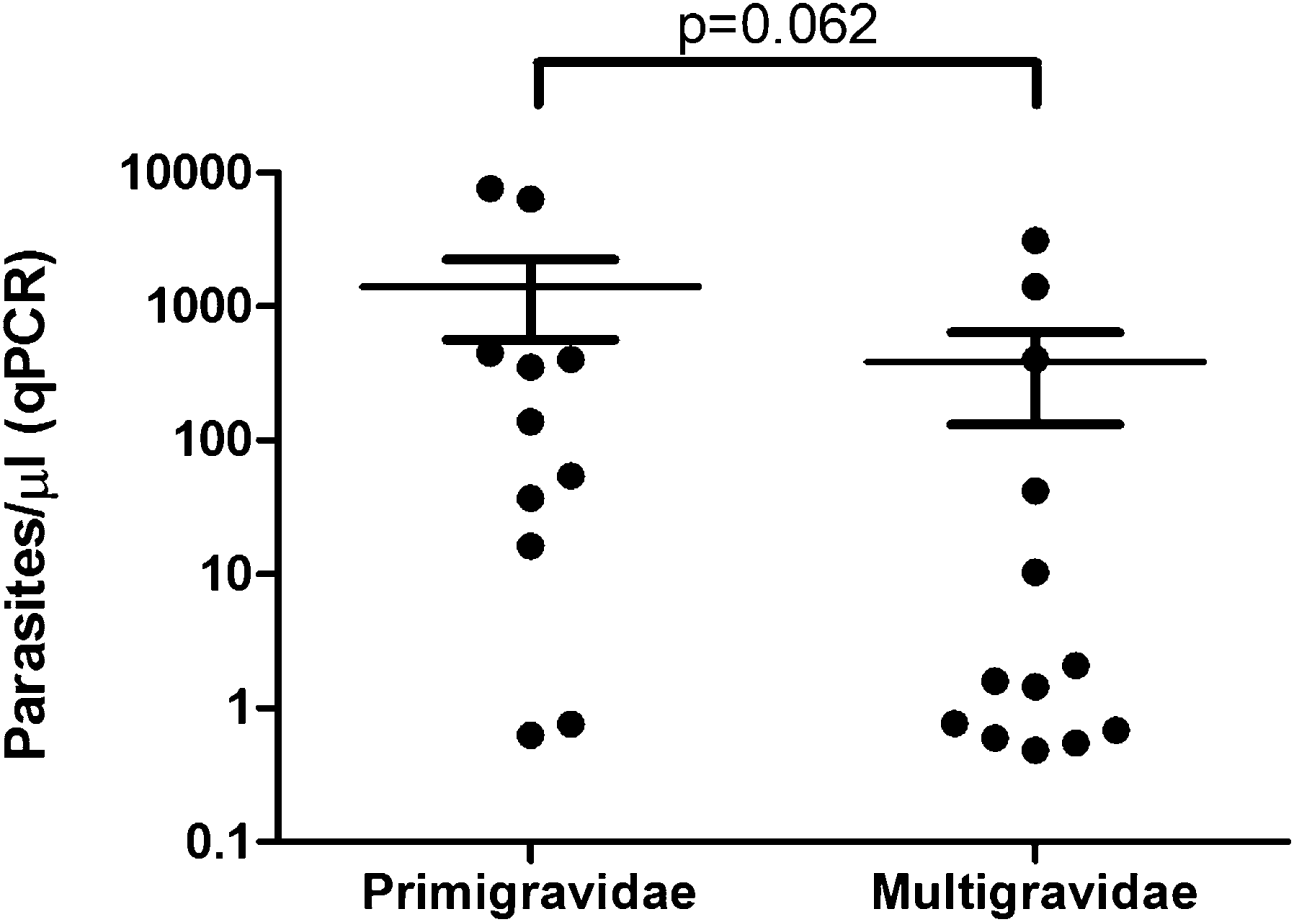
*Plasmodium falciparum* densities according to gravidity

Twenty-four women (12.3%) were confirmed as *P. falciparum* positive by qPCR (Table 2). Parasite densities tended to be lower in multigravidae than in primigravidae (Fig. 1). Low density *P. falciparum* infections (parasite densities below 10 parasites/µL) were more frequent among multigravidae (62% [8/13]) than among primigravidae (18% [2/11]; p = 0.047).

Compared to the uninfected women (Table 2), *P. falciparum*-infected women were significantly younger (24.9 vs. 27.6 years; p = 0.044) and in their first pregnancy (45.83 vs. 24.7%; p = 0.030). No associations between infection and education or occupation were found.

No statistically significant differences in terms of haemoglobin levels were found between the *P. falciparum*-infected (mean 10.89; range 9.2–13.7) and uninfected women (mean 11.26; range 7–16; Table 2). In total, 50.0% and 40.2% of the *P. falciparum*-infected and the uninfected women had anaemia, respectively (p = 0.364). Occurrence of fever was significantly more frequent among the infected women (14.3% vs. 1.8%; p = 0.019). No associations were found between *P. falciparum* infection and self-reported tuberculosis status, positive syphilis test, positive HIV test, and the use of ITNs (Table 2).

### Molecular markers of anti-malarial resistance

Of the 24 samples positive for *P. falciparum* by qPCR, 17 (70.8%) and 11 (45.8%) were successfully analysed for polymorphisms (*k13*, *pfmdr1*, *pfdhps* and *pfcrt*) and copy number (*pfpm2* and *pfmdr1*), respectively (Table 3). Sequencing analysis of all laboratory positive controls with known alleles revealed the presence of wild and mutant-type alleles of *k13*, *pfmdr1*, *pfcrt* and *pfdhps* polymorphisms. The estimated *pfpm2* and *pfmdr1* copy number for the laboratory positive controls (C4 and Dd2) were between 3 and 4 copies using SYBR^®^-based qPCR. The qPCR assays were specific to *P. falciparum* gDNA only, as no amplifications were noticed in negative controls (with water and human genomic DNA).

**Table 3:** Molecular markers of *P. falciparum* anti-malarial resistance

Genes	SNP	Wild-type	Mutant
*Pfmdr1* Multi-drug resistance	N86Y	8 (47.0%)	9 (52.9%)
	L155L (Syn)	16 (94.1%)	1 (5.9%)
	Y184F	7 (41.2%)	10 (58.8%)
	S1034C	17 (100.0%)	0 (0%)
	N1042D	17 (100.0%)	0 (0%)
	D1246Y	15 (88.2%)	2 (11.8%)
*Pfdhps* Sulfadoxine	S436A	12 (70.6%)	5 (29.4%)
	A437G	14 (82.3%)	3 (17.6%)
	K540E	17 (100.0%)	0 (0%)
	A581G	16 (94.1%)	1 (5.9%)
	A613S	15 (88.2%)	2 (11.8%)
*Pfcrt* Chloroquine	M74I	2 (11.8%)	15 (88.2%)
	N75E	2 (11.8%)	15 (88.2%)
	K76T	2 (11.8%)	15 (88.2%)
	I77I (Syn)	16 (94.1%)	1 (5.9%)
	I103I (Syn)	16 (94.1%)	1 (5.9%)
*K13* Artemisinin	V510V (Syn)	16 (94.1%)	1 (5.9%)

*SNP* single nucleotide polymorphisms, *Syn* synonymous mutation (no amino acid change)

The proportion of the *pfdhps* polymorphisms S436F, A437G, A581G and A613S described as associated with sulfadoxine resistance was 29.4% (5/17), 17.6% (3/17), 5.9% (1/17) and 11.8% (2/17), respectively. All the isolates had wild-type allele at K540E polymorphism of *pfdhps* gene. In the *pfcrt* gene, mutant alleles were found at codons M74I, N75E and K76T [88.2% (15/17)]. Two novel synonymous polymorphisms (I77I and I103I [5.9% (1/17)]) were observed in the *pfcrt* gene. A total of four polymorphisms were identified across the *pfmdr1* gene. The prevalence of N86Y, L155L, Y184F and D1246Y mutant alleles was 47.1% (8/17), 5.9% (1/17), 58.8% (10/17) and 11.8% (2/17), respectively. The other most frequent S1034C and N1042D polymorphisms of *pfmdr1* gene were not observed. The most frequent polymorphisms in *k13* observed previously in Cambodian isolates [25] were absent in the *P. falciparum* isolates analysed. However, a novel synonymous polymorphism V510V [5.9% (1/17)] was observed. All the isolates carried a single copy of *pfpm2* and *pfmdr1* genes.

## Discussion

This appears to be the first study conducted in Liberia assessing the prevalence of *P. falciparum* infection among pregnant women after the Ebola epidemic that led to the interruption of malaria control [10, 13]. This study found a 12.3% prevalence of *P. falciparum* (by qPCR) among pregnant women attending to their first ANC visit at a non-government hospital in Monrovia, and before administration of any IPTp-SP dose. Molecular analysis of genetic markers of anti-malarial resistance showed no compromised therapeutic efficacy of ACT, but suggests suboptimal prevention by SP provided as IPTp.

Young primigravidae had the highest burden of *P. falciparum* infection at first antenatal visit. Similarly, low density infections were more common in multigravidae than in primigravidae women. This difference was not due to increased use of ITNs among the multigravidae. Rather, this observation is in line with the concept that the lack of immunity to the antigenic variants presented by *P. falciparum* parasites that accumulate selectively in the placenta increases the risk of *P. falciparum* infection in primigravidae [48–50]. However, this prevalence is much lower than the prevalence observed in the national 2016 MIS among children aged 6–59 months (26.9% and 61.9% by RDT in urban and rural areas respectively) [6]. Use of ITN among our participants (28%) was slightly lower than was reported at the 2013 DHS (37%) [9] and at the 2016 MIS (35% in urban areas) [6]. Half of the *P. falciparum*-infected women (50.0%; see Table 2) had subclinical malaria (i.e. without fever, anaemia) and could sustain malaria transmission [51].

Genetic polymorphisms associated with parasite resistance to chloroquine and pyrimethamine were detected in rural Liberia as early as 1978 [52]. Three decades later, resistance to chloroquine and SP continued to rise [53]. In 2003, national policies endorsed ACT with artesunate–amodiaquine as first treatment option for *P. falciparum* uncomplicated malaria [5]. In this study, a high prevalence of K76T, N86Y, Y184F and D1246Y (*pfcrt* and *pfmdr1*) polymorphisms previously associated with resistance to chloroquine and to artesunate–amodiaquine (88.2, 47.1, 58.8 and 11.8%, respectively) was observed [29, 31, 32, 54, 55]. In contrast, prevalence of molecular markers of resistance to the sulfadoxine component of SP—considering 540 as a surrogate of quintuple mutants in Africa [56]—was relatively low, in accordance with molecular data obtained in the region [57]. The prevalence data of polymorphisms align with previously published data generated from the only recent study that collected pre-treatment isolates from children below 5 years of age, and reported occurrence of polymorphisms associated to current ACT used in Liberia [31]. In the present study, only one synonymous *k13* polymorphism was observed among the *P. falciparum* isolates tested, thus demonstrating the absence of genetic evidences of artemisinin resistance in the parasite population circulating in Monrovia.

To strengthen malaria control in post-Ebola Liberia, enhanced surveillance and community-tailored health promotion is deemed urgent. Given the significant investment in PCR equipment during the Ebola epidemic in Liberia, surveillance could be enhanced by improving capacities at multiple sentinel sites. Indeed, the addition of PCR-based diagnostics to detect *P. falciparum* was considered feasible and did not pose additional safety risks to staff during the Ebola outbreak [58]. Continuous surveillance of malaria at ANC could be useful to assess temporal trends in the burden of malaria, to promptly detect emergence of anti-malarial resistance, and to provide prevention, treatment and health education to the pregnant women at risk of malaria infection [59].

Accompanying these surveillance efforts with clinic and community-based health promotion could improve the success of control and preventive measures against malaria. As reported in a qualitative study conducted in Monrovia in parallel to this prevalence study, many pregnant women may see their access to malaria care impeded due to low perception of their increased risk to malaria and to avoid unofficial fees requested by health staff at government-run clinics [60]. As a result, many women may resort to traditional herbal medicine or may opt to self-medicate with substandard anti-malarials that are easily available in the local chemists. All these practices and attitudes could lead to the worsening of the symptomatology and prognosis of their malaria disease and to the appearance of resistances to LMoHSW-endorsed anti-malarial regimes, and must be considered in the planning of malaria control activities in Liberia.

### Limitations

An important limitation to consider is that the recruitment period took place from October to June. Hence, no participant was recruiting during the peak months of the rainy season, which may justify the relatively low prevalence observed. Another limitation is that this study was conducted in an institution that charges pregnant women for the first ANC, a factor that could have limited recruitment of certain women who may be more prone to exposure to malaria infection. The findings of this study might be extrapolated only to the group of women of low-middle class with access to employment and education.

Additionally, recall bias could have compromised participants’ self-reporting of previous use of malaria prevention and treatment. Potential effect of recall bias was minimized by intensive training on study specific procedures for data collection to the recruiting staff.

## Conclusion

In conclusion, *P. falciparum* infections are expected in at least one in every eight women attending first ANC at a non-Government-run facility in Monrovia and outside the peak of the rainy season. The risk of *P. falciparum* infection is higher among young primigravidae. No genetic evidence of resistance to artemisinins was found among the *P. falciparum* isolates tested. More epidemiological studies involving pregnant women in Liberia are needed to describe the risk of malaria in this highly susceptible group in other settings in the country, as well as to closely monitor the emergence of resistance to commonly used anti-malarials in-country.
